# Modular Approach to Spintronics

**DOI:** 10.1038/srep10571

**Published:** 2015-06-11

**Authors:** Kerem Yunus Camsari, Samiran Ganguly, Supriyo Datta

**Affiliations:** 1 School of Electrical and Computer Engineering, Purdue University, IN, 47907

## Abstract

There has been enormous progress in the last two decades, effectively combining spintronics and magnetics into a powerful force that is shaping the field of memory devices. New materials and phenomena continue to be discovered at an impressive rate, providing an ever-increasing set of building blocks that could be exploited in designing transistor-like functional devices of the future. The objective of this paper is to provide a quantitative foundation for this building block approach, so that new discoveries can be integrated into functional device concepts, quickly analyzed and critically evaluated. Through careful benchmarking against available theory and experiment we establish a set of elemental modules representing diverse materials and phenomena. These elemental modules can be integrated seamlessly to model composite devices involving both spintronic and nanomagnetic phenomena. We envision the library of modules to evolve both by incorporating new modules and by improving existing modules as the field progresses. The primary contribution of this paper is to establish the ground rules or protocols for a modular approach that can build a lasting bridge between materials scientists and circuit designers in the field of spintronics and nanomagnetics.

The developments of the last two decades have combined the distinct fields of spintronics and magnetism into a powerful force. Starting with the Giant Magnetoresistance (GMR) effect, the field has enjoyed continuous breakthroughs with new discoveries such as the large Tunneling Magnetoresistance (TMR) effect, spin-transfer-torque (STT) switching and more recently, high spin-orbit phenomena including the Giant Spin Hall Effect (GSHE) and Topological Insulators (TI)^1^,^2^,^3^,^4^,^5^,^6^.

Spintronic memory devices based on TMR and STT have already been commercialized while spintronic logic devices are still being actively explored^7^,^8^,^9^. New materials and phenomena continue to be discovered at an impressive rate which can be viewed as a continually expanding set of “building blocks’’ 10 for sophisticated functional devices.

The objective of this paper is to provide a quantitative foundation for this building block approach, so that new discoveries can be integrated into functional device concepts, quickly analyzed and critically evaluated.

Specifically, through careful comparison with available theory and experiment, we establish a set of elemental modules representing a diverse array of materials and phenomena (Fig. 1a). These elemental modules can be assembled seamlessly to model complex functional devices and experimental structures. Irrespective of their physical origin, the modules are formulated in terms of generalized voltages and currents. Complex circuits assembled using these modules can be solved by standard circuit techniques including standard solvers like SPICE.

The generalization of charge transport to include spin-transport in “circuits’’ was pioneered by Brataas *et al.* in Ref. 11,12, reformulated in the language of 4 × 4 matrices and extended to include magnetization dynamics by Ref. 13,14, further expanded and used extensively in Ref. 15, 16, 17, 18, 19. The primary contribution of this paper is to establish the ground rules or protocols for a modular approach that is founded on such spin-circuits by (1) expanding the existing list of circuit models and (2) enabling the introduction of new materials and phenomena to build a lasting bridge between materials scientists and circuit designers as both the fields of spintronics and nanomagnetics progress.

It is not at all obvious that complex spintronic phenomena can indeed be represented in terms of circuits. Consider for example, the basic device that started the field of spintronics, namely the Spin Valve, which is modeled as a series circuit of two interface modules (Fig. 1b) that represent an interface between a ferromagnet (FM) and a non-magnet (NM). The usual conductances of these interfaces do not depend on the direction of the magnetization of the FM layer. How can two such conductances in series result in a conductance that depends on the angle between the magnets, as required by the GMR effect ? What makes this possible is the representation of each interface not as a simple conductance, but as a 4 × 4 conductance matrix relating 4-component currents to 4-component voltages (1 for charge and 3 for spin). Spin circuits using these 4 × 4 conductances incorporate all the physics of spin accurately.

We extend this approach further to non-local spin valves (NLSV) (Fig. 1c), a ubiquitous setup that has been used in a wide range of experiments, such as the Hanle effect^20^, non-local spin-torque deposition^21^, voltage-controlled spin-precession^22^ and spin-injection to semiconductors^23^. The straightforward implementation of the NLSV reproduces equivalent results to those of other theoretical approaches that are well-established for such structures^24^.

We further show that recent experiments using the inverse GSHE to convert spin currents in NLSV into charge voltages^25^ are modeled simply by adding a new GSHE module to the existing NLSV circuit (Fig. 1c). This ability to integrate new phenomena onto an existing framework represents one of the most useful features of the modular approach.

A modular approach needs to supplement the transport modules described above with magnetic modules using voltage and current-like variables in order to allow seamless integration of spintronic and nanomagnetic phenomena. An example of this is the circuit module that simulates the Landau-Lifshitz-Gilbert equation describing the dynamics of magnets. We illustrate this module with two examples (Fig. 1d–e). The first is the STT-driven magnetic tunnel junction (MTJ) which is a well-known device currently under active development. The second is a proposal for integrating Read and Write device into a transistor-like device with gain and directionality that can be used to build logic circuits.

We envision the library of modules to evolve both by incorporating new modules and by improving existing modules as the field progresses. Open-source codes for these modules and example spin-circuits discussed in this paper are available at our website^26^.

*Outline of Paper:* In the rest of this paper, we analyze various spin-circuit examples of Fig. 1b–e, all composed of the elemental modules, introducing detailed descriptions of modules in the order they appear. Additionally, all the modules including their circuit descriptions are catalogued in the Supplementary Information. To demonstrate the simplicity and wide application range of our approach, we start with the simplest and earliest devices of spintronics (a) Spin valves and (b) Magnetic Tunnel Junctions (MTJ), then move onto (c) Non-local spin-valve structures in the context of high spin-orbit coupling phenomena, and (d) we analyze a functional spin-logic proposal, the Spin Switch.

## Spin Valves

Historically, the Giant Magnetoresistance (GMR) effect in spin valves has been a critical phenomenon in the development of spintronics. In this section, we analyze vaarious types of spin-valves using spin-circuits to benchmark our results with existing models and experiment with the purpose of benchmarking and illustrating the wide range of our framework.

Typically, a 2-current model^27^ starting from the Boltzmann equation treating up/down spins as independent current channels is used to model the GMR effect. The 2-current model can equivalently be expressed in terms of a one-charge and one-spin basis, instead of the up/down spin basis. The former can then be heuristically expanded to include all spin directions, x,y and z, making it a 4-current model^14^, and applied to general purpose circuits via modified nodal analysis^15^. As a result, the 4-current model can naturally take care of arbitrary directions of magnets, capturing angular MR as a function of the angle between the magnets in contrast to the 2-current model which only captures the collinear configurations (P or AP). Before we show detailed results regarding Spin Valves and MTJs, we describe the building blocks used in their assembly.

### Non-magnet (NM) Module

**Figure 2:**
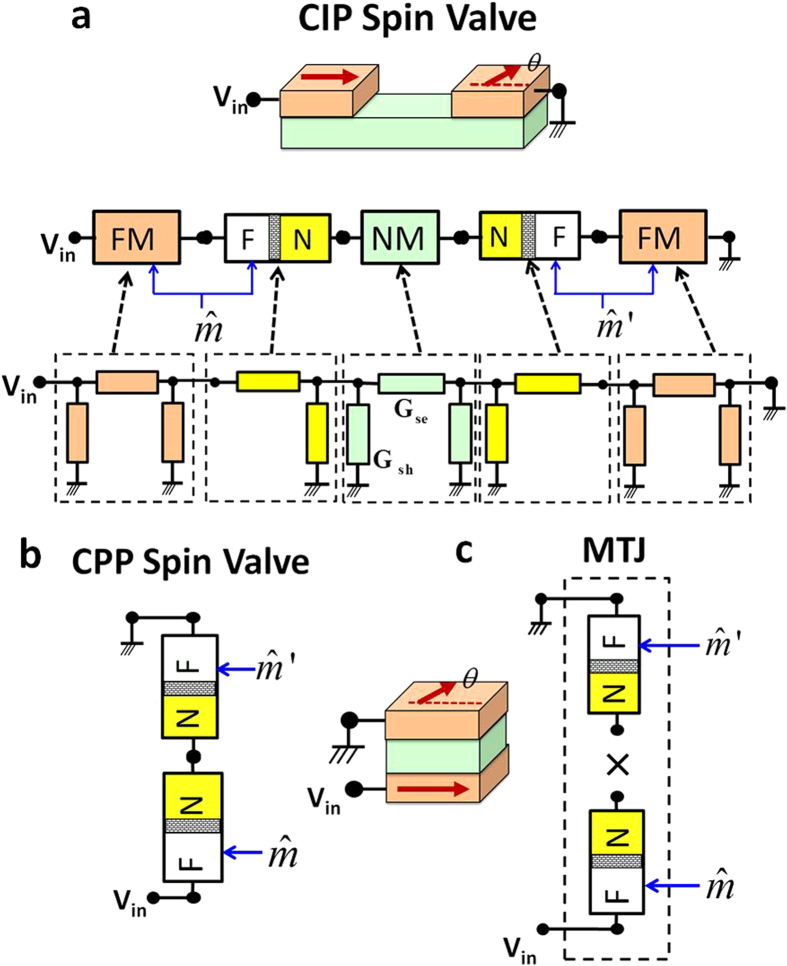
(**a**)—(**b**)Two types of spin valves, current-in-plane (CIP) and current perpendicular to plane (CPP) structures are assembled by FM, NM and FM–NM modules. (**c**) Magnetic tunnel junctions can be factored as a “product’’ of two FM–NM conductances^34^. The experimentally observed functional forms of angular magnetoresistance are both captured analytically in all three cases.

The non-magnet module describes a bulk, non-magnetic material with negligible spin-orbit coupling having two transport terminals and is modeled as a reciprocal Π-network containing a series and shunt conductance matrix (Fig. 2a). The series/shunt matrices are characterized by the resistivity (*ρ*), length (*L*), area (*A*) and spin-flip length (*λ*) of the non-magnetic material. The series conductance is given as:


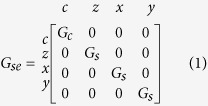


where *G*_*c*_ = *A*/(*ρL*) and *G*_*s*_ = *A*/(*ρλ*)csch(*L*/*λ*). The shunt conductance is given as:


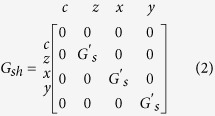


where *G*′_*s*_ = *A*/(*ρλ*)tanh(*L*/2*λ*). The shunt conductances account for the non-conservative spin-currents that decay over a few spin flip lengths, unlike charge currents. The charge column and row of the shunt conductance are zero, ensuring charge currents are always conserved.

It is important to note that the NM module presented here is not only valid for metals, but also for semiconductors such as silicon and graphene, as long as the transport is in the diffusive regime and well characterized by a conductivity and a spin-flip length.

### Ferromagnet (FM) Module

The FM module describes a bulk ferromagnet and is modeled as a reciprocal Π-network with a series and shunt conductance matrix (Fig. 2a), obtained from a spin-diffusion equation^14^,^54^. The series conductance for a +*z* directed magnet is given as:


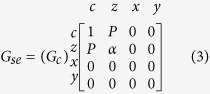


where *G*_*c*_ = *A*/(*ρL*) and *α* = *P*^2^+(1−*P*^2^)*L*/*λ* csch (*L*/*λ*). The shunt matrix for the FM:


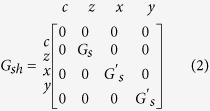


*G*_*s*_ = *A*/(*ρL*)(1–*P*^2^)(*L*/*λ*)tanh(*L*/2*λ*) and *G*′_*s*_ = *A*/(*ρλ*′) tanh(L/2*λ*′). *A* is area, *ρ* is resistivity, *L* is length, *P* is bulk magnet polarization, *λ* and *λ*' are longitudinal and transverse spin-flip lengths. Typically, *λ*' is much shorter than *λ* which is the spin-flip length along the magnetization direction.

*Rotation of FM and FM*–*NM*:The conductance matrices involving ferromagnets have been described for +*z* direction, in general, these matrices need to be expressed as a function of an arbitrary magnet direction, (*θ*,*ϕ*) through a basis transformation (*U*_*R*_ given in Supplementary Information):


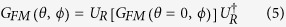


### FM—NM Interface Module

FM–NM module (Fig. 2a) represents the interface between a ferromagnet and a non-magnet, modeling the spin-currents through NM and FM layers. Spin currents in the transverse direction have an extremely short lifetime, decaying within a few monolayers of the magnet^12^. This requires the spin currents at the FM–NM interface to be modeled starting from a coherent transport theory. Here, we reformulate the experimentally established spin-mixing conductance theory^28^ pioneered by Brataas *et al.*^12^ in the language of the 4 × 4 conductance formalism^14^.

This module covers a wide range of interfaces from tunneling to ohmic contacts, and is characterized by an interface charge conductance *G*_0_, interfacial polarization (P), the real and imaginary spin-mixing conductances, and that can be obtained both from experiment and theory^12^. The series and shunt conductances for the FM–NM interface are given by:


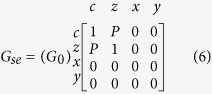



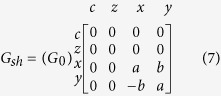


Note how the shunt conductance carries only an x and y current (transverse directions for a z-directed magnet) giving rise to the spin-torque current at the interface. This current is then supplied to an LLG solver as the spin-torque input.

*Asymmetry of FM*–*NM Interface:*The circuit description of the FM–NM interface has a shunt conductance only on its NM side and not on the FM side since the FM–NM interface is always preceded by a bulk FM region which does not carry any transverse spins. Therefore, no shunt conductance on the FM side is necessary. However, this assumption may break down for ultra-thin magnets where the FM is sandwiched by two NMs on either side (NM–FM–NM) where transverse spin-currents may travel through without getting completely absorbed by the magnet. In that case a more careful treatment of the interface conductances is necessary^12^.

*Magnetic Insulator*–*NM Interfaces:* Yttrium iron garnet (YIG), an insulating ferromagnet can potentially be very useful in spin devices^29^. When a spin current is incident to YIG, it acts as a spin sink absorbing a spin-torque, while it acts as an insulator to charge currents. Therefore, a YIG–NM interface can be modeled similar to a FM–NM interface, where the series conductance matrix becomes identically zero while the shunt conductances (and) are still of the order of the ballistic conductance ≈ (*q*^2^*M*)/(*h*), M being the number of modes in the NM^30^.

*Benchmark Results for Spin Valves:* Here, we demonstrate how various spin valve structures can be assembled using the FM, FM–NM and NM modules to benchmark spin-circuits with seminal results and stress how seemingly different structures are captured from a unified modular framework. Fig. 2 illustrates two structurally different types of spin valves, current-in-plane (CIP) that preceded the modern current-perpendicular-to-plane (CPP) spin valves. We show here that the angular magnetoresistance for CPP and CIP geometries are captured analytically from our formalism, reproducing experimental trends and earlier theory. The general results obtained for the conductances of spin valves here are 4 × 4 conductance matrices, however, we only consider the charge conductance (c,c) entry of the matrix) to relate to existing results.

*CPP Spin Valve:* The CPP spin-valve can be assembled as a series of two FM–NM interface conductances, one pointing along +z direction and one pointing in an arbitrary direction in the (z,x) plane:


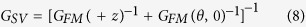


Assuming the terminals to have charge potentials only allows the series and shunt conductances to be added into a single conductance, *G*_*FM*−*NM*_ = *G*_*se*_ + *G*_*sh*_.

The (c,c) element of the *G*_*SV*_ matrix gives the angle-dependent charge conductance of the spin-valve. The angular magnetoresistance can analytically be obtained as:


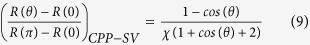


where *χ* = (*a*)/(1–*P*^2^)–1

This result has been first directly obtained from a Boltzmann equation based approach^35^ and later obtained by using the spin-mixing conductance concept^12^ with the assumption that the spacer resistance is negligible in the CPP configuration^55^, as we have also assumed in (Fig. 2b). Eq. (9) is known to reproduce the experimentally observed angular MR in CPP structures^35^,^36^.

*CIP Spin Valve:* It is experimentally and theoretically observed that the CIP geometry of spin valves has a different functional form compared to CPP^31^. We show that in the limit of high spacer resistances (*κ* = *R*_int._/*R*_*sp*._ ≈ 0), the circuit shown in Fig. 2a analytically reproduces the known angular dependence of CIP spin valves:


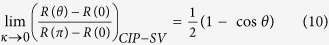


as observed for spin valves in CIP geometry^31^,^32^.

### Magnetic Tunnel Junction (MTJ)

Similar in structure to a CPP Spin-Valve, a MTJ is composed of two FM layers separated by an insulator that acts as a tunneling barrier and it has been modeled using the spin-circuit approach; using two FM–NM interfaces in series^33^. Here, we follow another approach where the conductance of the MTJ is the “product’’ of the two FM–NM interfaces^34^. Accordingly, this model needs to be included as a separate elemental module in our spin-circuit library, since the multiplication is not a standard circuit operation.


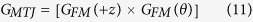


Similar to the CPP Spin-Valve, assuming that the terminals are “charge-driven’’ with no spin potentials, allows the FM–NM interface to be expressed as a single conductance, given by *G*_*FM*_–_*NM*_ = *G*_*sh*_ + *G*_*se*_.

Using the (c,c) entry of the total conductance matrix *G*_*MTJ*_, the angular MR is given by:


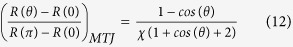


where *χ* = (2*P*^2^)/(1–*P*^2^).

Intuitively, the reason for multiplying the conductances is justified by considering that cascading two tunnel junctions of length *d*_1_ and *d*_2_ results in an exponential increase in the total resistance *R* ≈ exp(*d*_1_ + *d*_2_) = exp(*d*_1_)exp(*d*_2_). This argument was originally used to derive the TMR relation by Julliere^37^, and in the collinear configuration limit, Eq. (12) reduces to Julliere's formula, *G*_*P*_–*G*_*AP*_/*G*_*P*_ = 2*P*^2^/1–*P*^2^.

Note that the order of multiplication in Eq. (11) is significant for the full matrix conductance, however, it remains invariant for the charge conductance we have been discussing so far.

*Spin-Driven MTJs:* Recent experiments have shown that MTJs can be driven by pure spin currents in the absence of any charge currents^38^. So far, the MTJ conductances in the literature have been limited to charge-driven models where the spin and charge currents are expressed as a function of charge potentials at the terminals. The model we have described in this paper is a 4 × 4 matrix that can be spin-driven, however, only the first column is established by experiments and theoretical models^39^,^40^. Therefore, we leave the validation of the rest of the columns to future work.

*Numerical Validation:* The real power of our approach is that in general simplifying assumptions that we have made so far for the analytical calculations are not necessary. Complicated circuits such as Fig. 2a, that are tedious to solve analytically can be routinely simulated using standard circuit simulators^13^,^14^,^15^,^16^,^17^,^18^,^19^. In the Supplementary Information we show comparisons between experimentally obtained angular MR with the analytical results obtained in this section that are numerically validated using SPICE.

### Voltage-controlled spin precession

A milestone in the development of spintronics (Ref. 41) was the proposal of the so-called spin field-effect transistor, based on the voltage-controlled spin precession effect in a Rashba spin-orbit (RSO) channel^42^. Two decades later, Koo *et al.*^22^ experimentally demonstrated the proposed phenomenon in a non-local spin-valve geometry with a gate voltage that controlled the Rashba spin-orbit coupling of a 2DEG InAs channel using FM contacts. More recently, Chuang *et al.*^43^ demonstrated the same effect using specially engineered quantum point contacts (QPC) for efficient spin-injection into the channel. Here we view voltage-controlled spin-precession as a physical phenomenon or an ‘effect’ involving subtle spin-related properties and use it simply to illustrate the power of the modular approach.

**Figure 3:**
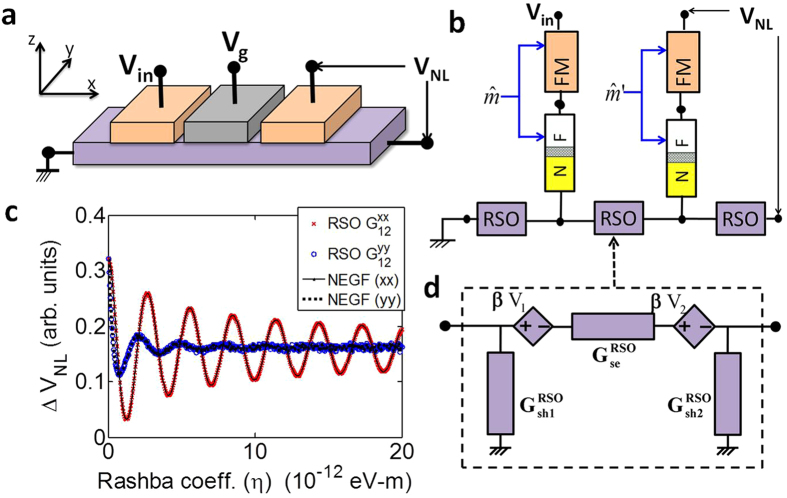
(**a**) Schematic of the experimental device that demonstrated the voltage controlled spin precession effect in a non-local spin valve^22^. (**b**) Spin-circuit representation of the device. (**c**) (y,y) and (x,x) entries of the 2D RSO conductance matrix as a function of the Rashba coefficient, benchmarked by the NEGF-based model^44^. (**d**) 4-component spin-circuit for RSO module.

A new effect like this can be analyzed simply by adding a new RSO module to the FM and FM–NM interface modules already described (Fig. 3). Instead of doing a full analysis of this device, we discuss the RSO module representing a channel that exhibits Rashba spin-orbit and benchmark it against a rigorous NEGF-based model^44^.

*Conductances to Spin-Circuits:* In order to obtain a 4-current circuit description of the RSO module, a coherent transport theory such as NEGF or Scattering Theory is needed. Following the NEGF-based prescription in^45^, the 4-component currents can be related to 4-component voltages in the following way:


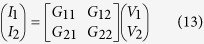


where *G*_*ij*_ are 4×4 conductances and *I,V* are 4×1 current vectors.

In general, universal sum rules and generalized Onsager relations restrict these conductance matrices, ensuring charge current conservation and micro-reversibility^45^,^46^. These restrictions exist for ordinary charge conductances (where *G*_*ij*_ are scalars) as well, however, charge conductances are always reciprocal (*G*_12_ = *G*_21_) in 2-Terminal devices, even in presence of magnetic fields and they always conserve charge currents (*G*_11_ + *G*_21_ = 0). None of these conditions automatically hold for spin-conductance matrices when *G*_*ij*_ are 4 × 4 matrices, as in Eq. (13). Spin-circuits may be non-reciprocal even in 2-Terminals and may not conserve spin-currents, even when the transport is coherent. We illustrate both of these effects in the context of the RSO module.

Once the conductance matrix description in Eq. (13) has been identified, a unique 4-component circuit can be constructed as shown in Fig. 3d.


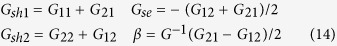


Note that a possible non-reciprocity in the conductance matrices (*G*_12_≠*G*_12_) requires dependent sources in the circuit model^45^.

### 1D RSO Module

Rasbha spin-orbit coupling in a semiconductor is well-described by the one-electron Hamiltonian^44^:


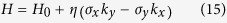


In a 1D-channel where transport is limited to the fundamental mode (*k*_*y*_ = 0), the Rashba term acts as an effective magnetic field similar to a Zeeman field, in the y-direction with magnitude |*ηk*_*x*_|. One crucial difference from the Zeeman field is, however this effective magnetic field is dependent on the momentum of the electron *k*_*x*_, changing sign for electrons traveling in opposite directions.

*Conductance Matrices for 1D RSO:* The conductance matrices in 1D (*k*_*y*_ = 0) for a ballistic RSO channel with NM leads at the ends are obtained using the formulation in^45^:


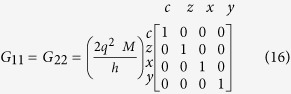


*G*_11_ and *G*_22_ are simply the ballistic interface conductances, due to the ideal NM leads. *G*_12_ and *G*_21_ read:


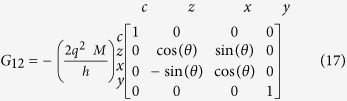



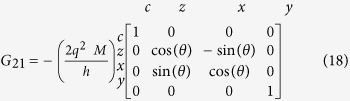


where the rotation angle is given by *θ* = 2m^*^ηL/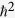, m^*^and *L* are effective mass and length of the channel and *η* is the Rashba coefficent and 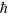 is the reduced Planck's constant^44^, in an effective mass approximation.

In the 1D model, *G*_12_ and *G*_21_ resemble rotation matrices around the y-axis (the effective field direction), but with opposite rotation angles, in accordance with the momentum dependent effective field.

*Ideal Interface Resistances:* The conductances of Eq. (13) are obtained from a coherent theory that places non-magnetic (NM) fictitious leads at the terminals, allowing a proper definition of terminal spin-currents and voltages. These NM leads introduce ballistic interface resistances proportional to the number of modes in the channel^47^ that needs to be subtracted when such coherent elements are added in series, as first pointed out by^48^.

*2D RSO:* Spin-transport in a 2D channel is more complicated than 1D, since electrons injected into the RSO channel can have different transverse momenta, not just limited to the fundamental mode (*k*_*y*_ = 0). As a result, the effective magnetic field direction changes with *k*_*y*_, and the time of flight increases with increasing *k*_*y*_ assuming periodic boundary condition at the walls, as was done in^44^.

Both of these effects can be accounted for by averaging 1D conductance matrices per transverse mode to obtain 2D conductance matrices, as we show in the Supplementary Information. The 2D conductance matrix obtained here is benchmarked against an NEGF-based model in^44^.

Figure. 3c shows the basic effect that was observed in^22^,^43^, the variation of *G*_12_(*x*,*x*) and *G*_12_(*y*,*y*) as a function of the RSO coefficient in the channel where (*x*,*x*) and (*y*,*y*) represent injector and detector magnets aligned in *x* and *y* directions respectively. Experimentally, when the injector and detector magnets were in the *x*-direction, a large oscillation in the non-local voltage was observed that was absent for *y*–*y* configuration of magnets^22^, as predicted by the corresponding RSO conductances shown in Fig. 3c.

### Giant Spin Hall Effect (GSHE)

The discovery of Giant Spin Hall Effect (GSHE) has generated a lot of interest due to its potential applications in spin-based memory and logic devices as a pure spin-current source^38^. Two important parameters characterizing GSHE are the spin Hall angle (*θ*_*SH*_) and the spin-flip length (*λ*). Here we introduce the GSHE module benchmarking a recent experiment^25^ that measured these parameters.

**Figure 4:**
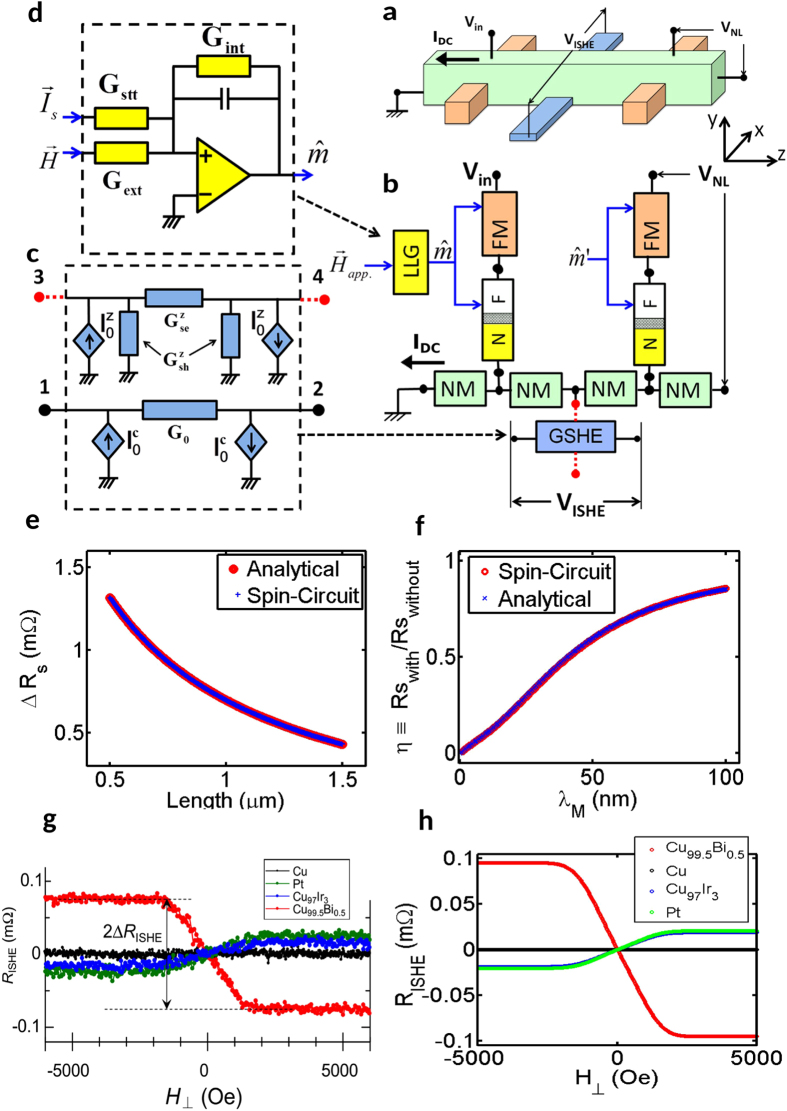
(**a**) Experimental setup for non-local spin valve with GSHE material^25^. (**b**) Spin-circuit implementation of the experimental structure. (**c**) Spin-circuit for the GSHE module. (**d**) Spin-circuit for the LLG solver module. (**e**) Non-local spin-resistance (*R*_*S*_ = *V*_*NL*_/*I*_*DC*_) as a function of channel length without the GSHE module. (**f**) Non-local spin-resistance as a function of spin-flip length of the GSHE module. (**g**) Experimental inverse spin Hall Effect (*R*_*ISHE*_ = *V*_*ISHE*_/*I*_*DC*_) resistance for different GSHE materials (**h**) Inverse spin Hall resistance obtained from the spin-circuit.

*Description of the Experiment:* The experimental setup and its spin-circuit model are shown in Fig. 4a–b. In the experiment, an external magnetic field is applied to change the magnetization direction of the injector magnet, controlling the polarization of injected spins into the NLSV. When the injector magnet points along +*z* direction, the spin current into the GSHE (CuBi) material that was embedded in the Cu channel induces a charge voltage along the +*x* direction (Fig. 4g). This voltage is proportional to the sign and magnitude of the spin Hall angle.

*NLSV without GSHE:* The experiment was first done without a (CuBi) bar in the middle for different (Cu) lengths and was theoretically calibrated by the Takahashi-Maekawa model^24^,^25^. The non-local spin-resistance obtained from the spin-circuit (Fig. 4b) exactly reproduces the Takahashi-Maekawa model using the dimensions and material parameters in the experiment (Fig. 4e). This step calibrates both the experiment and the spin-circuit without the GSHE material.

### GSHE Module

The GSHE module adopted in this paper is a 4-Terminal lumped circuit relating terminal currents to terminal voltages. It is derived from a modified spin-diffusion equation that takes the spin Hall angle as an experimental input^30^, and reproduces standard results for inverse and direct spin Hall effects^49^.

In typical GSHE experiments, a charge current flows through terminals 1–2 inducing a spin current that flows through terminals 3–4 (Fig. 4c). Accordingly, in the present version of the GSHE module, terminals 1–2 carry charge-currents only (solid in Fig. 4c) while terminals 3–4 carry a spin-current in one polarization direction (dashed in Fig. 4c). The direction of this polarization is given by the cross product of the spatial flow directions of the charge and spin currents^49^.

The circuit (Fig. 4c) between terminals 3–4 resembles the NM circuit, where the series/shunt conductances are given in terms of the conductivity (*ρ*), spin-flip length (*λ*), length (*L*), width (*W*), and thickness of the material (*t*):


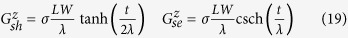


In addition, there are two controlled spin-current sources of opposite directions that generate the spin-current induced by the charge current flowing through terminals 1–2:


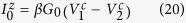


where *G*_0_ is the conductance of the film between terminal 1–2 and *β* is the spin Hall angle including the geometric factor:


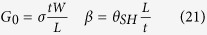


Similarly, the circuit between terminals 1–2 is composed of a series conductance and two controlled charge-current sources that are induced by the spin-currents flowing through terminals 3–4. The charge conductance is given by *G*_0_ and the current sources are given by:


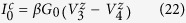


*NLSV with GSHE:* GSHE module is added to the NLSV as shown in Fig. 4b, leaving terminal 4 open to simulate the experimental conditions.

The non-local spin-resistance as a function of the spin-flip length of the GSHE material for a fixed spin Hall angle obtained from the spin-circuit exactly reproduces the analytical formulation that incorporates the GSHE into the Takahashi-Maekawa model described in the same experiment^25^, as shown in Fig. 4f.

Furthermore, the spin-circuit also reproduces the inverse spin Hall resistance that was measured in the experiment, for different materials (Fig. 4g,h). The numerical parameters used in the spin-circuit for these results are shown in the Supplementary Information.

*GSHE: Bulk or Interface Effect ?* It is important to note that the GSHE module is based on a modified spin-diffusion equation that assumes that the GSHE originates uniformly in the bulk of the material, thereby having symmetric spin-current sources for terminals 3–4. As the exact mechanism of the effect is better understood, the module can be modified such that the interfaces between the top and bottom surfaces are structurally asymmetric, leading to asymmetry in induced spin-current magnitudes.

*Connecting GSHE & RSO:* Both GSHE and RSO are high spin-orbit phenomena. However, in the case of RSO observed in semiconducting 2DEGs, spin-flip lengths (*λ*_*sf*_ ≈ 2*μ*m)^22^ are much longer compared to the spin-flip lengths (*λ*_*sf*_ ≈ 1 − 40 nm)^25^,^38^ in heavy metals exhibiting GSHE. Therefore, the spin-transport between the longitudinal terminals has not been considered in the GSHE module. If *λ*_*sf*_ was comparable to *L* in the current direction, the spin-circuit for GSHE would need to incorporate the spin-transport between terminals 1–2, and its conductance matrices *G*_12_ and *G*_21_ would incorporate rotation components similar to the RSO module.

### LLG Solver Module

In the experiment, a magnetic field in the perpendicular direction was used to reorient the injector magnet to +*z* direction. This step is included in the spin-circuit by having an LLG solver module which takes the magnetic field as an input and provides the magnetization direction as an output which is then routed to the FM and FM–NM interface modules.

The LLG module is a circuit that solves the Landau-Lifshitz-Gilbert equation to obtain the time-dependent magnetization dynamics in presence of magnetic fields and spin-currents. There are several possible ways of implementing the LLG solver as a circuit^16^,^50^. The LLG solver implemented here is an op-amp circuit that integrates 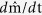 with an initial condition 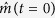. The internal fields of the magnet appear as a feedback loop in the circuit (Fig. 4d) while spin-currents and external fields are added as external inputs, mirroring the physics of magnetization dynamics. At present, the LLG solver does not consider thermal noise which can be added as a random voltage source to the circuit in future versions.

### Functional spin device: Spin Switch

**Figure 5:**
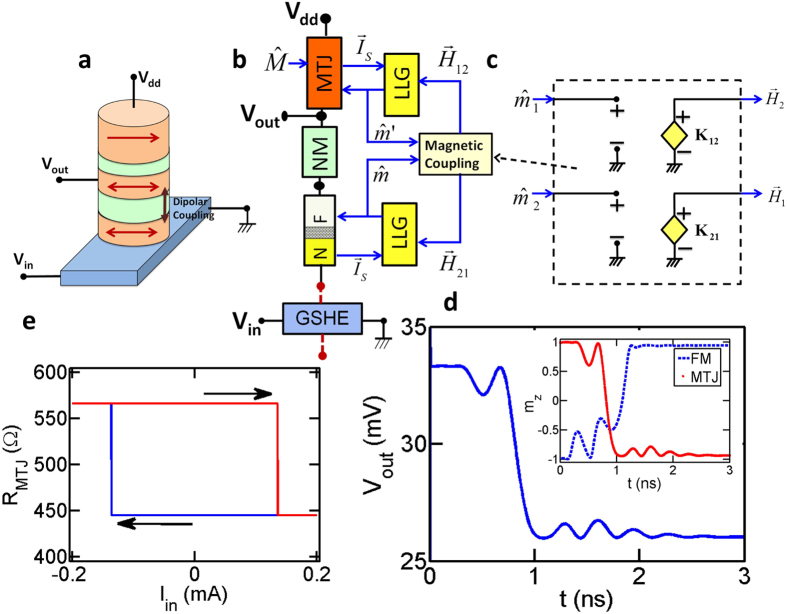
(**a**) Illustrative functional spin-logic device: Spin-Switch^8^. (**b**) Spin-circuit implementation of Spin-Switch. (**c**) Spin-circuit of magnetic coupling module. (**d**) Transient characteristics for an input current greater than critical switching (*I*_*in*_ = 2*I*_*c*_) (Inset) Time-dependent example of dipolar switching between the FM and free layer of the MTJ. (**e**) Steady state characteristics of the device as a function of charge current in GSHE.

An example of a functional spin-logic device is the recently proposed Spin Switch in Ref. 8 (Fig. 5a,b). This device is similar to the structure demonstrated in Ref. 38 and couples a GSHE-driven magnet with the free layer of an MTJ through dipolar interaction.

This device consists of two stages: a Write stage made of a thin magnet (GSHE-driven) and a Read stage (MTJ). These two stages are electrically isolated by a high resistivity non-magnet (NM) (Fig. 5b) but magnetically coupled through the dipolar interaction. This structure exhibits electrical isolation and gain that are crucial for building larger circuits^8^.

This device can be assembled using GSHE, MTJ, FM–NM Interface, NM, LLG, and the Magnetic Coupling modules (Fig 5b).

### Magnetic Coupling Module

Magnetic Coupling module represents the magnetic interaction between a pair of magnets to be used for both exchange and dipolar type interactions. The inputs and outputs to the module are the magnetization of the two magnets and the two magnetic fields they exert on each other (Fig. 5c). The coupling coefficients that determines these magnetic fields are computed using the dimensions and material properties of the magnets and they are fixed for a given geometry^51^. The details of the magnetic modules are given in the Supplementary Information.

*Device Behavior and Characteristics:* A single-switching event for the Spin-Switch is shown in Fig. 5d where a charge current (*I*_*in*_) flows in the GSHE, inducing a spin-current into the in-plane FM reversing it from –*z* to +*z* direction through spin-torque action. Due to the dipolar interaction, the free layer of the MTJ switches from +*z* to −*z*, changing the resistance of the MTJ thereby changing the output voltage (inset Fig. 5d). The transient simulation allows a quantitative analysis of the switching delay and energy dissipation that is normally not accessible to experiments probing steady-state switching characteristics.

By sweeping the input voltage and running transient simulation for each voltage value, we also construct the full switching characteristics of the device (Fig. 5e) that provides the critical switching current. The parameters for these simulations are given in the Supplementary Information.

### Summary

In summary we believe this paper establishes a quantitative foundation for a building block approach to spintronics by (1) identifying a basic set of elementary modules, (2) defining both transport and magnetic blocks in terms of voltage and current-like variables, (3) benchmarking each module against available theoretical models and experimental data, and (4) showing that many of the seminal results in the field are captured using circuits built out of these modules. The modular approach allows (1) Independent improvement of individual modules and (2) Expansion of the library to include new phenomena and materials as they are discovered. Examples of future extensions include Topological Insulators (TI), Voltage-Controlled Magnetic Anisotropy (VCMA) and spin-pumping. Open-source codes of the elemental modules and spin-circuits analyzed in this paper are available at our website^26^.

## Additional Information

**How to cite this article**: Camsari, K. Y. *et al.* Modular Approach to Spintronics. *Sci. Rep.* **5**, 10571; doi: 10.1038/srep10571 (2015).

## Supplementary Material

Supplementary Information

## Figures and Tables

**Figure 1:**
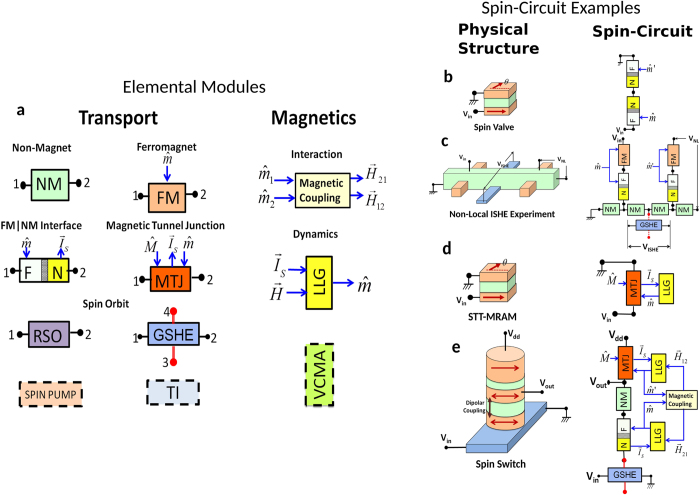
(**a**) The modules in the circuit library come in two broad categories, transport blocks (TB) based on the physics of transport and magnetic blocks (MB) based on the physics of magnetism. Solid blocks are presented in detail in the manuscript, dashed blocks (Spin pumping, Topological Insulators, Voltage-Controlled Magnetic Anisotropy) are envisioned modules for the future. Illustrative spin-circuits: (**b**) Spin-Valve (**c**) Non-local spin valve with inverse spin Hall effect (**d**) Spin-transfer-torque MRAM (**e**) Spin Switch: A proposed spin-logic device^8^.
