# Supplementary material for: Modular Approach to Spintronics
Source: Sci Rep. 2015 Jun 11;5:10571. doi: 10.1038/srep10571 (PMC4464157; doi:10.1038/srep10571)
Supplement: Supplementary Information [file srep10571-s1.pdf]

Modular Approach To Spintronics: Supplementary Information

Kerem Yunus Camsari,* Samiran Ganguly, and Supriyo Datta
School of Electrical and Computer Engineering, Purdue University, IN, 47907

I. MODULES

This section gives a detailed overview of all the modules mentioned in the main paper.

A. Non-Magnet(NM)

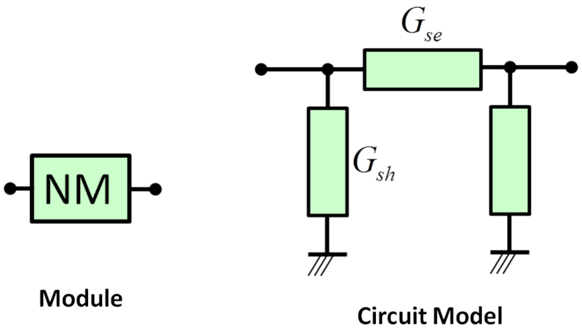

FIG. 1. Circuit model for Non-Magnet module

$$G_{se} = \begin{array}{c} \\ c \\ z \\ x \\ y \end{array} \begin{array}{c} \begin{array}{cccc} c & z & x & y \end{array} \\ \begin{bmatrix} G_c & 0 & 0 & 0 \\ 0 & G_s & 0 & 0 \\ 0 & 0 & G_s & 0 \\ 0 & 0 & 0 & G_s \end{bmatrix} \end{array} \quad G_{sh} = \begin{array}{c} \\ c \\ z \\ x \\ y \end{array} \begin{array}{c} \begin{array}{cccc} c & z & x & y \end{array} \\ \begin{bmatrix} 0 & 0 & 0 & 0 \\ 0 & G'_s & 0 & 0 \\ 0 & 0 & G'_s & 0 \\ 0 & 0 & 0 & G'_s \end{bmatrix} \end{array} \tag{1}$$

where $G_c = A/(\rho L) \quad G_s = A/(\rho\lambda)\text{csch}(L/\lambda) \quad G'_s = A/(\rho\lambda)\tanh(L/2\lambda)$.

Parameter	Symbol	Units
Length	L	m
Area	A	m^2
Resistivity	ρ	Ω-m
Spin-flip Length	λ	m

* kcamsari@purdue.edu

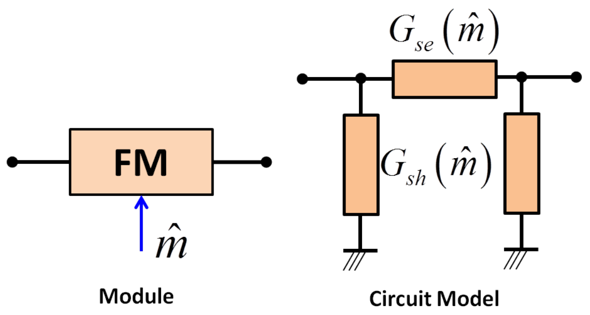

FIG. 2. Circuit model for Ferromagnet module

B. Ferromagnet(FM)

$$G_{se} = G_c \begin{array}{c} \\ c \\ z \\ x \\ y \end{array} \begin{array}{c} \begin{array}{cccc} c & z & x & y \end{array} \\ \begin{bmatrix} 1 & P & 0 & 0 \\ P & \alpha & 0 & 0 \\ 0 & 0 & 0 & 0 \\ 0 & 0 & 0 & 0 \end{bmatrix} \end{array} \quad G_{sh} = \begin{array}{c} \\ c \\ z \\ x \\ y \end{array} \begin{array}{c} \begin{array}{cccc} c & z & x & y \end{array} \\ \begin{bmatrix} 0 & 0 & 0 & 0 \\ 0 & G_s & 0 & 0 \\ 0 & 0 & G'_s & 0 \\ 0 & 0 & 0 & G'_s \end{bmatrix} \end{array} \tag{2}$$

where $G_c = A/(\rho L)$, $G_s = A/(\rho L)(1-P^2)(L/\lambda)\tanh(L/2\lambda)$, $G'_s = A/(\rho\lambda')\tanh(L/2\lambda')$ and $\alpha = P^2 + (1-P^2)L/\lambda\ \mathrm{csch}(L/\lambda)$.

**Parameter**	**Symbol**	**Units**
Length	L	m
Area	A	m^2
Resistivity	ρ	Ω-m
Polarization	P	-
Longitudinal Spin-flip Length	λ	m
Transverse Spin-flip Length	λ'	m

C. FM−NM Interface

$$G_{se} = (G_0) \begin{array}{c} \\ c \\ z \\ x \\ y \end{array} \begin{array}{c} \begin{array}{cccc} c & z & x & y \end{array} \\ \begin{bmatrix} 1 & P & 0 & 0 \\ P & 1 & 0 & 0 \\ 0 & 0 & 0 & 0 \\ 0 & 0 & 0 & 0 \end{bmatrix} \end{array} \quad G_{sh} = (G_0) \begin{array}{c} \\ c \\ z \\ x \\ y \end{array} \begin{array}{c} \begin{array}{cccc} c & z & x & y \end{array} \\ \begin{bmatrix} 0 & 0 & 0 & 0 \\ 0 & 0 & 0 & 0 \\ 0 & 0 & a & b \\ 0 & 0 & -b & a \end{bmatrix} \end{array} \tag{3}$$

**Parameter**	**Symbol**	**Units**
Conductance	G_0	S
Polarization	P	-
In-plane torque coeff.	a	-
Out-of-plane torque coeff.	b	-

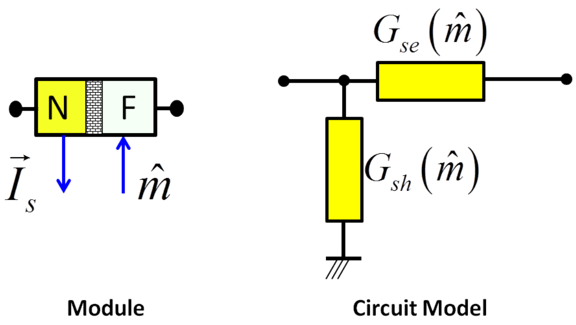

FIG. 3. Circuit model for FM−NM module

D. Rotation matrix

$$[G(\theta,\phi)]^{FM} = U_R\left[G^{FM}(\theta=0,\phi)\right]U_R^{\dagger} \tag{4}$$

$$U_R = \left[\begin{array}{c|cccc} & c & z & x & y \\ \hline c & 1 & 0 & 0 & 0 \\ z & 0 & \cos(\theta) & \sin(\theta)\cos(\phi) & \sin(\theta)\sin(\phi) \\ x & 0 & -\sin(\theta)\cos(\phi) & \cos(\theta)+\sin^2(\phi)(1-\cos(\theta)) & -\sin(\phi)\cos(\phi)(1-\cos(\theta)) \\ y & 0 & -\sin(\theta)\sin(\phi) & -\sin(\phi)\cos(\phi)(1-\cos(\theta)) & \cos(\theta)+\cos^2(\phi)(1-\cos(\theta)) \end{array}\right] \tag{5}$$

E. Rashba Spin-Orbit Coupling (1D)

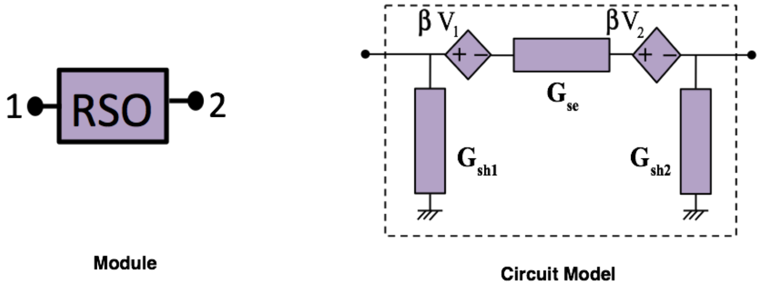

FIG. 4. Circuit model for RSO module

$$\begin{pmatrix} I_1 \\ I_2 \end{pmatrix} = \begin{bmatrix} G_{11} & G_{12} \\ G_{21} & G_{22} \end{bmatrix} \begin{pmatrix} V_1 \\ V_2 \end{pmatrix} \tag{6}$$

$$\begin{aligned} G_{sh1} &= G_{11}+G_{21} \quad & G &= -(G_{12}+G_{21})/2 \\ G_{sh2} &= G_{22}+G12 \quad & \beta &= G^{-1}(G_{21}-G_{12})/2 \end{aligned} \tag{7}$$

$$G_{11} = G_{22} = \left(\frac{2q^2\ M}{h}\right) \begin{array}{c} \\ c \\ z \\ x \\ y \end{array} \begin{array}{c} \begin{array}{cccc} c & z & x & y \end{array} \\ \begin{bmatrix} 1 & 0 & 0 & 0 \\ 0 & 1 & 0 & 0 \\ 0 & 0 & 1 & 0 \\ 0 & 0 & 0 & 1 \end{bmatrix} \end{array} \tag{8}$$

$$G_{12} = -\left(\frac{2q^2\ M}{h}\right) \begin{array}{c} \\ c \\ z \\ x \\ y \end{array} \begin{array}{c} \begin{array}{cccc} c & z & x & y \end{array} \\ \begin{bmatrix} 1 & 0 & 0 & 0 \\ 0 & \cos(\theta) & \sin(\theta) & 0 \\ 0 & -\sin(\theta) & \cos(\theta) & 0 \\ 0 & 0 & 0 & 1 \end{bmatrix} \end{array} \quad G_{21} = -\left(\frac{2q^2\ M}{h}\right) \begin{array}{c} \\ c \\ z \\ x \\ y \end{array} \begin{array}{c} \begin{array}{cccc} c & z & x & y \end{array} \\ \begin{bmatrix} 1 & 0 & 0 & 0 \\ 0 & \cos(\theta) & -\sin(\theta) & 0 \\ 0 & \sin(\theta) & \cos(\theta) & 0 \\ 0 & 0 & 0 & 1 \end{bmatrix} \end{array} \tag{9}$$

$$\text{where } \theta = \frac{2m^*\eta L}{\hbar^2}$$

F. Rashba Spin-Orbit Coupling (2D)

G_{11} and G_{22} remain unchanged for mode dependent conductances. $G_{12}(\theta, \phi)$ and $G_{21}(\theta, \phi)$ read:

$$G_{12} = -\left(\frac{2q^2\ M}{h}\right) \begin{array}{c} \\ c \\ z \\ x \\ y \end{array} \begin{array}{c} \begin{array}{cccc} c & z & x & y \end{array} \\ \begin{bmatrix} 1 & 0 & 0 & 0 \\ 0 & \cos(\theta') & \sin(\theta')\cos(\phi) & -\sin(\theta')\sin(\phi) \\ 0 & -\sin(\theta')\cos(\phi) & \cos(\theta')\cos(\phi)^2 + 1 - \cos(\phi)^2 & -\sin(\phi)\cos(\phi)(\cos(\theta') - 1) \\ 0 & \sin(\theta')\sin(\phi) & -\sin(\phi)\cos(\phi)(\cos(\theta') - 1) & \cos(\theta') - \cos(\theta')\cos^2(\phi) + \cos^2(\phi) \end{bmatrix} \end{array}$$

$$\text{where } \theta' = \frac{\theta}{\cos(\phi)} \text{ and } \phi = \tan^{-1}\left(\frac{k_y}{k_x}\right)$$

$$G_{21} = -\left(\frac{2q^2\ M}{h}\right) \begin{array}{c} \\ c \\ z \\ x \\ y \end{array} \begin{array}{c} \begin{array}{cccc} c & z & x & y \end{array} \\ \begin{bmatrix} 1 & 0 & 0 & 0 \\ 0 & \cos(\theta') & -\sin(\theta')\cos(\phi) & -\sin(\theta')\sin(\phi) \\ 0 & \sin(\theta')\cos(\phi) & \cos(\theta')\cos(\phi)^2 + 1 - \cos(\phi)^2 & -\sin(\phi)\cos(\phi)(\cos(\theta') - 1) \\ 0 & -\sin(\theta')\sin(\phi) & -\sin(\phi)\cos(\phi)(\cos(\theta') - 1) & \cos(\theta') - \cos(\theta')\cos^2(\phi) + \cos^2(\phi) \end{bmatrix} \end{array}$$

$$\text{where } \theta' = \frac{\theta}{\cos(\phi)} \text{ and } \phi = \tan^{-1}\left(\frac{k_y}{k_x}\right)$$

The mode-dependent conductances need to be added in order to get a single 2D conductance:

$$G_{21}^{2D} = \frac{\int_{-\pi/2}^{\pi/2} G_{21}(\phi)\ d\phi}{\pi} \tag{10}$$

**Parameter**	**Symbol**	**Units**
Length	L	m
Effective Mass	m^*	kg
Rashba coeff.	η	eV-m
No. of modes	M	-

G. Giant Spin Hall Effect

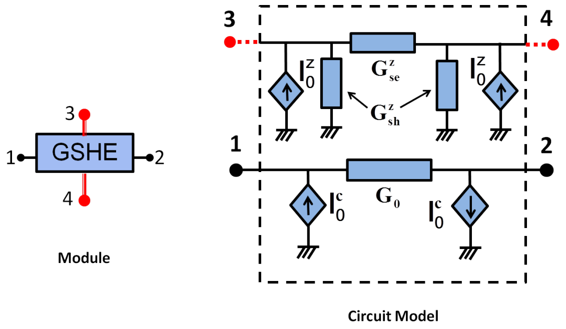

FIG. 5. Circuit model for GSHE module

$$G^z_{sh} = \sigma\frac{LW}{\lambda}\tanh(\frac{t}{2\lambda}) \quad G^z_{se} = \sigma\frac{LW}{\lambda}\text{csch}(\frac{t}{\lambda}) \tag{11}$$

$$I^z_0 = \beta G_0(V^c_1 - V^c_2) \tag{12}$$

$$G_0 = \sigma\frac{tW}{L} \quad \beta = \theta_{SH}\frac{L}{t} \tag{13}$$

$$I^c_0 = \beta G_0(V^z_3 - V^z_4) \tag{14}$$

**Parameter**	**Symbol**	**Units**
Spin Hall angle	θ	-
Length	L	m
Width	W	m
Resistivity	ρ	Ω-m
Thickness	t	m
Spin-flip Length	λ	m

H. Magnetic Tunnel Junction

The details of the MTJ model obtained by the matrix multiplication of the conductance matrices are given in [1]. In this paper, we have only considered the low-bias angular dependence of the charge conductance of the MTJ to draw comparisons with Spin Valves.

$$G^{cc}_{21} = G^{cc}_{12} = 1 + P_M P_m \cos(\theta) \tag{15}$$

where G_{12} and G_{21} correspond to different orders of multiplication for the interface conductances. The multiplication is commutative for the charge conductance. θ is the relative angle between $\hat{M}$ and $\hat{m}$.

**Parameter**	**Symbol**	**Units**
Conductance	G_0	S
Polarization (Fixed FM)	P_M	$-$
Polarization (Free FM)	P_m	$-$

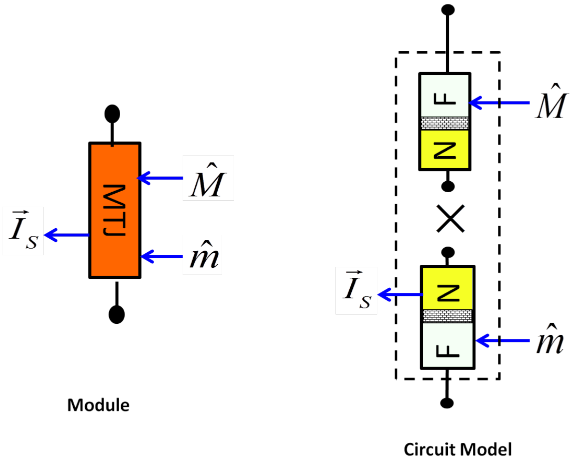

FIG. 6. Circuit model for MTJ module

I. LLG Solver

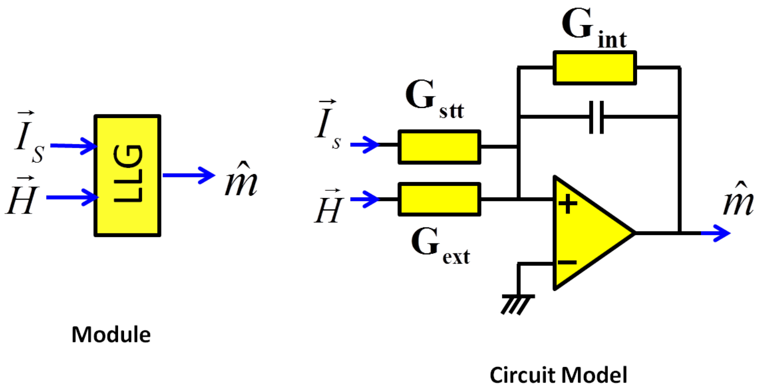

FIG. 7. Circuit model for LLG module

LLG equation (normalized units):

$$\left(\frac{1+\alpha^2}{\gamma H_k}\right)\frac{d\hat{m}}{dt} = -\hat{m}\times\vec{h} - \alpha\ \hat{m}\times\hat{m}\times\vec{h} - \hat{m}\times\hat{m}\times\vec{i_s} + \alpha\ \hat{m}\times\vec{i_s} \tag{16}$$

LLG is solved by an opamp based integrator circuit. At the + node of the opamp the nodal equation is:

$$C\frac{d\hat{m}}{dt} = G_{int}\ \hat{m} + G_{ext}\vec{H}_{ext} + G_{stt}\vec{i}_s \tag{17}$$

Since the nodal equation has the same form, the voltage appearing at the output of the opamp is the solution of the LLG equation. The internal/self magnetic field is constructed from the output of the opamp as a feedback using the matrix relation given below:

$$\begin{Bmatrix} \vec{H}_x \\ \vec{H}_y \\ \vec{H}_z \end{Bmatrix}_{int} = \begin{bmatrix} K_x & 0 & 0 \\ 0 & K_y & 0 \\ 0 & 0 & K_z \end{bmatrix} \begin{Bmatrix} \hat{m}_x \\ \hat{m}_y \\ \hat{m}_z \end{Bmatrix} \tag{18}$$

The vector products appearing on the RHS of the LLG equation are carried out by the 3×3 conductance matrices which can be viewed as operators acting on the magnetic field and spin torque current. These are given as:

$$G_{int} = X(1 + \alpha X)\frac{K_{int}}{H_k} \quad G_{stt} = X(X + \alpha)\frac{1}{qN_sH_k} \quad G_{ext} = X(1 + \alpha X)\frac{1}{H_k} \tag{19}$$

where X is the cross product operator with $\hat{m}$:

$$X = \begin{bmatrix} 0 & -m_z & m_y \\ m_z & 0 & -m_x \\ -m_y & m_x & 0 \end{bmatrix} \tag{20}$$

As an example, for an in-plane magnet, whose axis is in $\hat{z}$ and out-of-plane is in $\hat{x}$, $K_z = 1, K_y = 0, K_x = -4\pi M_s/H_k$. For an PMA magnet with out-of-plane in $\hat{x}$, $K_x = 1, K_y = 0, K_z = 0$. Note that the normalization needed for the LLG equation are handled by these operator conductances.

**Parameter**	**Symbol**	**Units**
Saturation Magnetization	M_s	emu/cc
Damping coeff.	α	-
Anisotropy field strength	H_k	Oe
Internal field coeff.	K_x, K_y, K_z	-
Area of cross section	A	m^2
Thickness	t	m

J. Magnetic Coupling

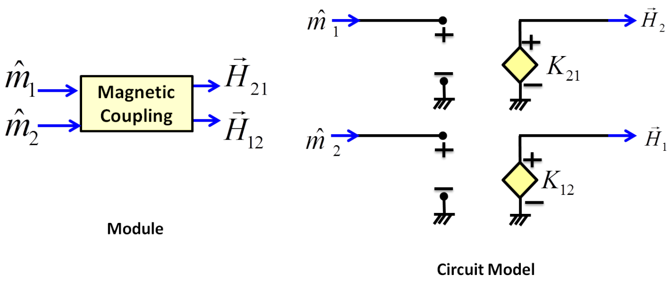

FIG. 8. Circuit model for Magnetic Coupling module

The magnetic field between two magnets j and i is given by:

$$\begin{Bmatrix} \overrightarrow{H}_x \\ \overrightarrow{H}_y \\ \overrightarrow{H}_z \end{Bmatrix}_j = \begin{bmatrix} K_{xx} & K_{xy} & K_{xz} \\ K_{yx} & K_{yy} & K_{yz} \\ K_{zx} & K_{zy} & K_{zz} \end{bmatrix}_{ji} \begin{Bmatrix} \hat{m}_x \\ \hat{m}_y \\ \hat{m}_z \end{Bmatrix}_i \tag{21}$$

These matrix elements $K_{\alpha\beta}$ can be adjusted to generate a magnetic interaction of certain type: Dipolar and exchange coupling, are calculated from magnetostatic equations or exchange integrals. The inputs for these calculations could be material properties like saturation magnetization, electronic bandstructure (for exchange integral), geometrical dimensions and separation of the two magnets. The detailed computation of the dipolar interaction used in this paper are given in [2].

These matrix elements need to be precomputed for a given set of magnets and provided to the module as parameters while the spin circuit is setup. The inputs for the module are the magnetization of the two magnets and output are the magnetic fields between them. The circuit is implemented using two VCVS whose gain coefficients are the coupling matrices, as shown in the circuit diagram.

**Parameter**	**Symbol**	**Units**
Coupling matrix between 1 and 2	K_{12}	Oe
Coupling matrix between 2 and 1	K_{21}	Oe

II. ANGULAR MAGNETORESISTANCE

Here we provide experimental benchmarks of angular MR obtained from spin-circuits for (1) MTJ (2) CPP spin-valve, and (3) CIP spin-valve, showing the functional form of MR as a function of relative angle (θ) of the two magnets.

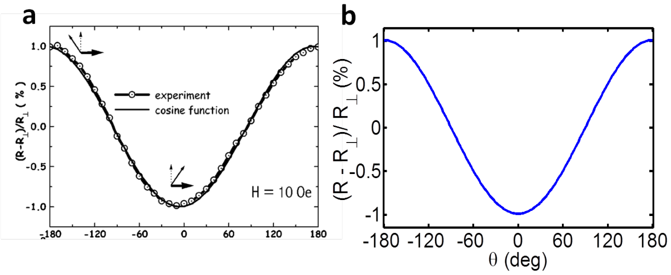

FIG. 9. (a) Experimental MR of an MTJ with respect to (θ) reproduced with permission from [3] (b) Functional form of TMR using P=0.1

.

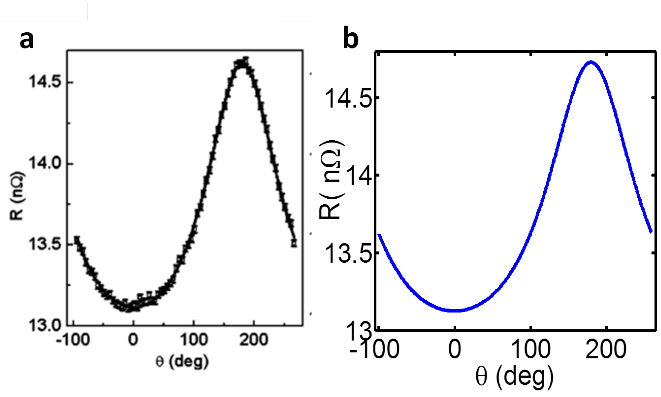

FIG. 10. (a) Experimental MR of a CPP spin-valve with respect to (θ) reproduced with permission from [4] (b) Functional form of CPP-GMR using and $R_P = 13.25n\Omega$, $a = 2.8$, $P = 0.33$

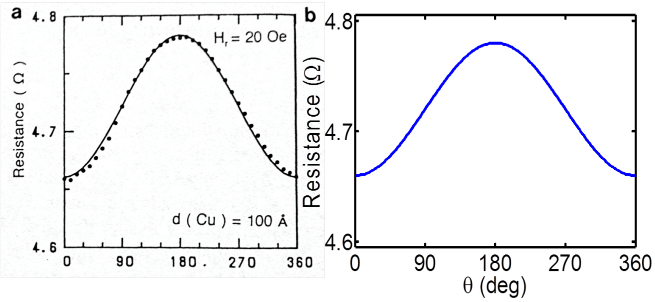

FIG. 11. (a) Experimental MR of a CIP spin-valve with respect to (θ) reproduced with permission from [5] (b) Functional form of CIP-GMR using and $R_P = 4.66\Omega$, $R_{AP} = 4.78\Omega$

III. INVERSE SPIN HALL EFFECT IN THE NON-LOCAL SPIN VALVE GEOMETRY

A. Without the GSHE module

The table below provides the parameters for the elemental modules to reproduce the analytical Takahashi-Maekawa formula, that are fit to reproduce the experimental results for three different channel lengths in [6].

**Parameters**	**FM (both identical)**	**FM-NM (both identical)**	**NM1 (left-most)**	**NM2=NM3 (middle regions)**	**NM4 (right-most)**
Area	1e-14 m^2	1e-14 m^2	1e-14 m^2	1e-14 m^2	1e-14 m^2
Length	1e-7 m	N/A	1e-5 m (does not affect R_s)	Length (x-axis)	1e-5 m (does not affect R_s)
Polarization	0.23	0.11	N/A	N/A	N/A
Spin-flip length	5e-9 m (longitudinal) 5e-10 m (transverse)	N/A	1.3e-6 m	1.3e-6 m	1.3e-6 m
Resistivity	1.9e-7 $\Omega - \mathrm{m}$	N/A	1.5e-8 $\Omega - \mathrm{m}$	1.5e-8 $\Omega - \mathrm{m}$	1.5e-8 $\Omega - \mathrm{m}$
Resistance per Area	0.5e-15 $\Omega - \mathrm{m}^2$	N/A	N/A	N/A	N/A
Spin-mixing conductances	N/A	In-plane (a)=1 Out-of-plane (b)=0	N/A	N/A	N/A

B. With the GSHE module

The parameters for the simulation of the spin circuit without the GSHE (above) remain the same for the circuit with the GSHE module. In addition to those parameters, the parameters used for the GSHE are:

**Spin-flip length**	**Resistivity**	**Spin-Hall Angle**	**Effective Length**	**Width**	**Thickness**	**Effective Area**
λ (x-axis)	11.3e-8 $\Omega - \mathrm{m}$ (for $Cu_{99.5}Bi_{0.5}$)	-0.12 (for $Cu_{99.5}Bi_{0.5}$)	100e-9 m	250e-9 m	20e-9 m	100e-9 m $\times$ 250e-9 m

IV. SPIN SWITCH

Material parameters and dimensions used for simulation of the spin switch device are given below. The NM is used an insulator by making its resistivity very high ($\rho \approx \infty$).

**GSHE**					
Spin-flip length	Resistivity	Spin-Hall angle	Length	Width	Thickness
1 nm	1e-6 Ohm-m	0.3	80 nm	100 nm	2 nm
**FM-NM Interface**					
Conductance (G_0LW)	Polarization	In-plane coeff. (a)	Out-of-plane coeff. (b)		
40 S	0.5	1	0		
**LLG (FM and MTJ)**					
Saturation Magnetization (M_s)	Damping	Coercivity	Area	Thickness	
700 emu/cc	0.01	40 Oe	80 nm $\times$ 100 nm	2 nm	
**MTJ**					
Conductance (G0)	Polarization (fixed layer)	Polarization (free-layer)			
1e-3 S	0.5	0.5			
**Dipolar Coupling**					
K_{xx}= 35.5 Oe	K_{yy}=$-$19.9 Oe	K_{zz}=$-$15.7 Oe			

[1] K. Y. Camsari, S. Ganguly, D. Datta, and S. Datta, International Electron Devices Meeting (2014).
[2] V. Diep, C.-C. Lin, R. Faria, A. Verma, J. Appenzeller, Z. Chen, and S. Datta, (in preparation) (2014).
[3] H. Jaffrès, D. Lacour, F. Nguyen Van Dau, J. Briatico, F. Petroff, and A. Vaurès, Physical Review B **64**, 064427 (2001).
[4] S. Urazhdin, R. Loloee, and W. P. Pratt, Physical Review B **71**, 100401 (2005).
[5] T. Okuyama, H. Yamamoto, and T. Shinjo, Journal of Magnetism and Magnetic Materials **113**, 79 (1992).
[6] Y. Niimi, Y. Kawanishi, D. H. Wei, C. Deranlot, H. X. Yang, M. Chshiev, T. Valet, A. Fert, and Y. Otani, Physical Review Letters **109**, 156602 (2012).
